# Color, Healthcare and Bioethics

**DOI:** 10.5195/jmla.2026.2319

**Published:** 2026-01-01

**Authors:** Endah Fitriasari

**Affiliations:** 1 Program Studi Ilmu Keperawatan, STIKes Maluku Husada, Jl. Lintas Seram. Kairatu SBB

In *Color, Healthcare and Bioethics*, Henk ten Have presents a groundbreaking and profoundly interdisciplinary examination of the role of color in medicine and bioethics. Far from being a mere aesthetic detail, color is revealed as a critical element in diagnosis, treatment, and our broader understanding of health. Ten Have expertly weaves together scientific, philosophical, and ethical threads to demonstrate how color, in its physiological and symbolic forms, holds deep implications for healthcare. The book's central contribution is its argument that color is not a passive visual cue but an **active force**, influencing human psychology, societal structures, and moral judgments particularly in discussions of race and equity within healthcare systems. This work offers an unprecedented perspective that challenges the traditional, often narrow, view of bioethics.

## 1. Introduction: Color, Healthcare, and Bioethics

The introductory chapter meticulously frames color as a neglected but powerful influence in both healthcare and bioethics. Ten Have argues that while objective data is paramount in medicine, color's subjective experience and cultural weight carry immense, often unseen, significance in diagnostic and therapeutic settings. By discussing the historical and cultural connotations of color, he makes a compelling case for its inclusion in serious academic discourse. This chapter successfully re-situates healthcare, moving beyond a purely scientific paradigm to one that acknowledges the human element and the non-obvious ways that visual phenomena shape medical practice and ethical debate. It serves as a powerful call to action, urging a more holistic and nuanced approach to bioethics.

## 2. The Nature of Color

Chapter two delves into the complex nature of color itself, bridging philosophy and science. Ten Have guides the reader from classical understandings, such as those of Newton, to modern neurophysiological theories, which posit color as a construct of the visual system rather than an inherent property of objects. This discussion is foundational, as it establishes color as a phenomenon that exists at the intersection of the objective and subjective. The most compelling aspect of this chapter is its exploration of phenomenological realism, which portrays color not as an abstract quality but as a relational property integral to the lived, immediate world a concept that resonates throughout the book and strengthens its central thesis.

## 3. The Power of Color

This chapter transitions to the psychological and emotional impact of color. Ten Have masterfully integrates scientific research with insights from the arts to illustrate how color affects human emotions and behavior. He cites research showing that specific colors, like red and blue, evoke predictable psychological responses that have direct implications for patient environments and therapeutic outcomes. For example, blue often promotes a sense of calm, while red can signify urgency or danger. By incorporating the views of artists such as Henri Matisse and Wassily Kandinsky, the book highlights color as a potent emotional and psychological force, making this chapter a valuable resource for anyone involved in healthcare design or patient-centered care.

## 4. Color and Healthcare

Here, the book provides a robust and practical examination of color's application in medical settings. Ten Have offers concrete examples of how doctors use visual cues such as cyanosis (blue skin) or jaundice (yellow skin) for critical diagnoses. Beyond diagnosis, the chapter explores the therapeutic uses of color, from color therapy to the creation of healing hospital environments. This emphasis on tangible applications of color in medicine constitutes one of the book's most significant contributions, demonstrating how a deeper understanding of color can tangibly enhance both diagnostic accuracy and patient well-being.

## 5. Color and Bioethics

This chapter represents the ethical core of the book. Ten Have confronts the ethical dimensions of color head-on, particularly its entanglement with race and social justice. He meticulously deconstructs how color, especially in the binary of black and white, has been used historically to create racial hierarchies and how this legacy persists in contemporary healthcare disparities. By examining the subtle and not-so-subtle racial biases in treatment, the book issues a powerful call for bioethics to expand its framework to address these deeply ingrained prejudices. This timely analysis provides a moral compass for navigating the complex social justice issues that plague healthcare today.

## 6. A Colorful Bioethics

In the concluding chapter, Ten Have proposes a truly visionary paradigm shift: the integration of color as a central component of bioethical discourse. He argues that color should be recognized as a key element in ethical deliberations, especially in matters of race, identity, and healthcare access. This call for a “colorful bioethics” challenges the field's traditional, rigid frameworks, advocating for a more inclusive understanding that embraces the emotional, cultural, and symbolic power of color. It is a thought-provoking conclusion that invites scholars and practitioners alike to re-examine how bioethics can better address the nuanced and deeply human realities of healthcare.

## COMPARISON WITH SIMILAR PUBLICATIONS

*Color, Healthcare and Bioethics* distinguishes itself from other works by introducing the under-explored concept of color into the bioethics and medical fields. Ten Have's work broadens the conversation to include a more holistic, philosophical, and aesthetic dimension of color itself. His integration of color theory into ethical considerations is unprecedented. While many bioethics texts rely on objective rationality, Ten Have's approach is more expansive, inviting cultural, emotional, and aesthetic perspectives into the dialogue, making the book particularly relevant for scholars interested in the intersection of healthcare, race, and ethics.

## POTENTIAL READERSHIP

This book is an essential read for a diverse audience, including bioethicists, medical professionals, sociologists, philosophers, and students of medicine and healthcare. Its interdisciplinary nature also makes it valuable for those interested in color theory, race studies, and cultural analysis. Given its focus on social justice, the book will also resonate with a wider audience, including activists and policy reformers working to address racial inequalities in healthcare.

## CONCLUSION

*Color, Healthcare and Bioethics* is a groundbreaking work that successfully bridges philosophy, bioethics, and color theory. Ten Have's comprehensive examination of color's power and ethical implications provides an innovative and timely contribution to the field. The book not only challenges conventional thinking but also offers practical insights into how color influences health, behavior, and moral decision-making. It is essential reading for any-one seeking a deeper understanding of the profound role of color in healthcare and the ethical issues surrounding it.

